# Influence of Environmental Risk Exposure on the Determinants of COVID-19 Booster Vaccination in an Urban Thai Population

**DOI:** 10.3390/ijerph21060745

**Published:** 2024-06-06

**Authors:** Weerawat Ounsaneha, Orapin Laosee, Cheerawit Rattanapan

**Affiliations:** 1 Faculty of Science and Technology, Valaya Alongkorn Rajabhat University under the Royal Patronage Pathumthani Province, Klong Nuang, Klong Luang, Pathumthani 13180, Thailand; weerawat@vru.ac.th; 2 ASEAN Institute for Health Development, Mahidol University, Salaya, Phutthamonthon, Nakhon Pathom 73710, Thailand; orapin.lao@mahidol.edu

**Keywords:** environmental risk exposure, COVID-19 vaccine booster, post-pandemic

## Abstract

This study aimed to identify the influence of environmental risk exposure levels on the predictive factors of COVID-19 booster dose vaccination in an urban Thai population in the post-pandemic era. Six study locations, including the three provinces with the highest environmental risk levels and the three provinces with the lowest environmental risk levels, were selected by calculating the environmental risk exposure indexes. Participants from the capital district of each province were chosen via the simple random sampling technique and interviewed using a structured questionnaire. A total of 1315 individuals were included in a sample in this study, and the best predictors of booster dose vaccination were determined using multiple regression analysis. The results showed that a high level of environmental risk exposure occurred in the provinces with a high number of total days exceeding the limits set for PM10 and high rates of mortality for lung cancer. The number of COVID-19 booster vaccinations given amount to 43.4% of the population during the post-COVID-19 pandemic period. Our multivariate analysis indicated that individuals in the working age group (≥25 years old); those with higher education (diploma degree and above); full-time employment (government and private sectors); those with high monthly incomes (≥USD144.1); and those in areas with the lowest risk level of environmental exposure significantly contributed to the number of booster dose vaccinations given during the post-pandemic period. To summarize, the rate of COVID-19 booster dose vaccination acceptance in Thailand was influenced by socio-economic factors with environmental concerns. These findings improve our understating of both the global pandemic and how environmental exposure affects behavioral change patterns and could improve the effectiveness of post-pandemic management.

## 1. Introduction

Urbanization and modernization have quickly become extensive, pushed by industrialization and economic development [1,2]. Additionally, urbanization has led to a high demand for energy and natural resources, accompanied by environmental pollution [3,4]. Thunis’s report [5] identified that air quality is a serious environmental problem in urban locations. In terms of environmental conditions, various influencing factors, including population density, space area, and poor urban ventilation quality, have strongly affected the health outcomes of city residents [6,7]. By 2050, around 66.2% of the Thai population will be located in urbanized areas [8]. Jareemit et al. [7] identified that the health burden in Thai urban locations is caused by the low rate of air ventilation in these areas, which is due to a lack of urban planning, design, and land use management. The limitations of the development interventions to reduce health inequality in the urban setting were found to be because of unclear scientific knowledge of the environmental exposure measures and on the interaction between space and society [9].

In periods of disease outbreaks, environmental propulsion should be studied to identify the transmission severity of the disease. Environmental factors can determine the level and identify the spread of many pathogens [10]. Moriyama et al. [11] found that environmental determinants influence host susceptibility by modulating airway defense processes, affecting respiratory virus transmission. Various studies have reported that environmental factors, such as temperature [12], population density [13], air pollution [14], sea air masses [15], etc., may have a significant impact on the number of confirmed COVID-19 cases. Specifically, the social interactions within populations in cities with adverse environmental factors, including low wind speeds and high air pollution, can lead to rapid virus transmission and a high number of COVID-19 fatalities [16,17].

SARS-CoV-2 vaccination is one effective approach to protecting against mortality and preventing its transmission in populations [18]. The ongoing administration of COVID-19 booster dose vaccines is the most efficient mechanism to protect against COVID-19, based on the recommendations of public health institutions [19]. People’s hesitancy to receive booster dose vaccination can be attributed to many issues, such as political change and reduced trust in public health guidelines [20,21]. Immunity has been found to be provided by second-dose vaccination six months after the first vaccination, and reported cases of COVID-19 reinfection have decreased [22]. Presently, an ongoing number of booster doses against COVID-19 have been recommended as an effective preventive measure for vaccinated adults. Annual and bi-annual booster dose vaccinations in high-risk groups and the general population have been suggested by the WHO [23].

Based on the present prevention and control policy for COVID-19, using the new generation of COVID-19 vaccination has been recommended by public health institutions, as well as by the Thai government [24]. The number of booster dose vaccinations administered for COVID-19 in Thailand during the high endemic peak of COVID-19 demonstrated that most of the population (69.4%) were vaccinated with COVID-19 booster doses [25]. Intawong et al. [26] highlighted the significant potential of the third and fourth booster doses to prevent against infections from the delta and omicron variants of COVID-19 found in the Thai population. Notably, an up-to-date bivalent vaccination consisting of two different strains of the virus was recommended, as it presents better protection against COVID-19 transmission than the latest variants [27]. Hence, increasing booster dose coverage should be supported as a Thai national policy during any COVID-19 outbreak.

Urban populations have a high-risk exposure to air pollutants and a high risk of diseases and premature mortality [28]. Evidence from reports has shown a link between air pollution and the occurrence of cardiovascular diseases [29], cancer [30], and death in urban locations [31]. Specifically, air pollution is an ongoing experience for residents in the northern and central parts of Thailand. Poapongsakorn et al. [32] identified that 29,000 Thai residents died due to respiratory diseases, stroke, and cardiovascular diseases caused by air pollution exposure. Moreover, with COVID-19’s spread, the high number of deaths in cities was caused by extremely high levels of environmental pollution present, due to their low ventilation and a high concentration of air pollutants [17]. Hence, understanding environmental risk exposure at the city level is the one key elements of epidemic prevention and control, indicating the population’s exposure to infectious diseases.

Ounsanha et al. [25] identified a high rate of booster dose vaccination in a Thai population located in the area with the highest risk of environmental exposure during the omicron variant outbreak, with a trend of increasing numbers of COVID-19 booster vaccinations during a high-peak outbreak [26]. Recognizing the gap in the findings of existing publications, this ongoing research aimed to identify the influence of environmental risk exposure levels on the pattern of booster dose vaccination in a Thai population after the COVID-19 outbreak period. Using a survey, residents in the areas of highest and lowest environmental exposure in Thailand were selected for comparison to update our understanding of the factors influencing people’s decision on whether to receive booster vaccinations against COVID-19 after the pandemic crisis and to propose recommendations for the Thai government using the concept of environmental risk exposure.

## 2. Materials and Methods

### 2.1. Study Area

As 20 different provinces had the most and least COVID-19 cases in Thailand [33], our study areas were located throughout the country. According to Coccia’s method for calculating the environmental risk exposure index [17], data on environmental factors from 2022 including the days exceeding the total daily limits set for PM10 (days), the wind speed (km/h), the density of population (inhabitants per km^2^), and the mortality rates due to lung cancer (100,000 people) were collected from the responsible government organizations [34,35,36,37]. After completing data collection, 25th, 50th, and 75th percentile values for each factor in each province were separated into four groups: group I (>25th percentile), group II (between the 25th and 50th percentile), group III (between the 50th and 75th), and group IV (above the 75th percentile). The points for each factor in each province were defined as 0 to 3 items: item 1 = 0, item 2 = 1, item 3 = 2, and item 4 = 3. Finally, the level of environmental risk exposure (*LERE*) was calculated by the modifying data of these environmental factors using Equation (1) below.

(1) $$LERE=\frac{\left[EF1\left(S_{k1}\right)+EF2\left(S_{k2}\right)+EF3\left(S_{k3}\right)+EF4(S_{k4})\right]}{12}$$

where

*LERE* = level of environmental risk exposure
*EF*1, 2, 3, 4 = environmental factors
*S_k_* = score of each factor (*EF*) *i* between 0 and 3.

The level of environmental risk exposure ranged from 0 to 1. The highest risk levels of environmental exposure in different areas merit scores close to 1, while areas with scores close to 0 had the lowest levels of environmental exposure to COVID-19.

### 2.2. Study Population and Procedure

A cross-sectional study using face-to-face interviews was collected on the residents living in areas with the highest risk levels (three provinces) and lowest risk levels (three provinces) of environmental exposure to COVID-19, determined from related environmental risk exposure index assessments. The data were collected using simple random sampling methods between 15 March and 15 May 2023. A 95% confidence interval and 20% acceptable error were used to estimate the sample size of participants in this study. With a nonresponse rate of 20%, the 1315 individuals were equally recruited from both size areas of the study settings. The inclusion criteria for participant recruitment were participants being >18 years, Thai citizens, and having resided in the study area for more than six months. Research assistants introduced and delivered the objective, background, and benefit of the study, as well as a consent form for the participants’ confidentiality and privacy. These assistants were trained, over one-day meetings, to clarify, standardize, and improve the quality of the data collection process. The Committee for Research Ethics (Social Science), Faculty of Social Science and Humanities, Mahidol University, granted this study the certificate approval number 2023/018.1402 and its MU-SSIRB number was 2023/009(B1). All procedures of this research were conducted according to the Declaration of Helsinki on the ethical principles for medical research.

### 2.3. Measurement

The tool for measurement in this study was designed based on related questionnaires [38,39,40,41] which were then translated to Thai. The pilot test of questionnaire modification was conducted on 30 residents in Nakhon Pathum Province to determine the validity and reliability, and this test generated a Cronbach’s alpha coefficient of 0.70. The final questionnaire in used this study consisted of four parts with forty-five questions. The first part of socio-demographic factors included age, sex, marital status, education level, occupation, monthly income, COVID-19 history, and insurance. The second part, which was on COVID-19’s impact, comprised a total of ten questions investigating three items, including causal attribution, emotion, and resilience using Likert scales ranging from 1 = strongly disagree, 2 = disagree, 3 = not sure, 4 = agree, to 5 = strongly disagree. The third part used Likert scales ranging from 1 = strongly disagree to 5 = strongly agree and covered environmental concerns, which were divided into two items: (1) environmental attitude (seven questions) and (2) environmental behavior related to infectious prevention and control (eight questions). The fourth part regarding COVID-19 preventive behavior contained 10 Likert-scale questions. In terms of the outcome variable, reception of the booster vaccination was verified and recorded by a formal vaccine certificate from the Thai government using a mobile phone application [25].

### 2.4. Statistical Analysis

Descriptive statistics, including percentages, means, or medians, standard deviations, or quartile deviations, were used to characterize the sociodemographic factors determined in this study. In terms of data distribution, the Kolmogorov–Smirnov test was used to examine the normality. The Chi-square test was used for the comparison of highest and lowest areas of environmental risk exposure with respect to the booster dose vaccination rate against COVID-19. Multivariate logistic regression was identified as a strong predictor of booster dose vaccination, with *p* < 0.05, and the adjusted odds ratio was calculated as 95% of the confidence interval. The statistical software IBM-SPSS, Version 21 was used to compute this statistical analysis.

## 3. Results

Table 1 presents the risk profile of environment exposure in the 10 provinces in Thailand with the highest numbers of COVID-19 cases and the 10 provinces with the lowest numbers of COVID-19 cases on 1 October 2022 during the post-COVID-19 period. The risk level rank of 0 to 1 from the environmental exposure assessment identified the highest risk of exposure as 1.00, as per Coccia [25]. The environmental exposure areas used as the sample area in this study were ranked in terms of the third highest and lowest risk levels of environmental exposure out of the ten provinces. The result showed that the studied provinces with the highest environmental risk exposure were Bangkok, Samut Prakan, and Samut Sakhon, and those with the lowest environmental risk exposure were Mae Hong Son, Mukdahan, and Phichit. These were determined in order to identify the impact of environmental risk exposure on COVID-19 vaccine boosters in a Thai population after the pandemic crisis.

The populations (1315) in this study consisted of two study areas: 601 (45.7%) participants lived in areas with a low level of environmental risk exposure area and 714 (54.3%) lived in areas with a high level of environmental risk exposure area after the COVID-19 outbreak (Table 2). In total, 42.9 and 57.1% of participants were male and female, respectively. The age ranges of the population in both areas were equal. In terms of education, 40.5% of the participants attended secondary school. The most common marital status (67.0%) was single/divorced. The most common occupation and monthly income for the population was general employee (34.4%) and an income of more than 287 USD (52.9%), respectively. In total, 809 (61.5%) subjects were COVID-19 positive, with family members (30.4%) as the primary source of COVID-19 transmission. In total, 82.1% of the population had health insurance. A total of 1315 subjects were analyzed, and the demographics obtained, including their age, education, marital status, occupation, monthly income, COVID-19 status, source of COVID-19, and health insurance, were statistically significantly differences between those located in a low risk of environmental exposure and those located in a high risk of environmental exposure (*p*-value < 0.05).

Table 3 shows the number of COVID-19 booster vaccines administered in low- and high-risk areas of environmental exposure in Thailand. The results demonstrated that most of the populations in both areas received their third dose of a COVID-19 vaccine (43.4%) and COVID-19 booster vaccines (63.7%). When analyzing the acceptance of COVID-19 vaccination within the study location, the number of vaccines received and the booster dose received significantly differed between the populations in the areas with low and high environmental exposure risks (*p*-value < 0.05).

Table 4 shows the levels of COVID-19 impact, environmental concerns, and COVID-19 prevention behavior in populations in Thailand with the lowest and highest risks of environmental exposure to COVID-19. In terms of COVID-19’s impact, three categories, causal attribution, emotion, and resilience, were investigated in both study areas. The results showed that low levels of causal attribution (63.7%), emotion (61.1%), and resilience (66.5%) were observed across both study areas. In addition, no statistically significant differences were noted in these three levels between both study areas’ populations (*p*-value < 0.05). In the context of environmental concerns, the levels of two categories, environmental attitude and environmental behavior, were determined in both study areas. The results implied that environmental attitudes in both study areas’ populations were at good levels (71.2%) without significant differences (*p*-value < 0.05). In terms of environmental behavior, a poor level of environmental behavior (59.7%) was found in areas with a high risk of environmental exposure. This was in contrast to the result of areas with a low risk of environmental exposure; a good environmental behavior level (61.9%) was present in this population. 

A statistically significant difference was observed in the environmental behavior of populations between study areas (*p*-value < 0.05). COVID-19 preventive behavior was considered low by 57.0% of the population in areas with a high risk of environmental exposure, while 50.4% of the population in areas with a low risk of environmental exposure exhibited good environmental behavior. This corresponded to our statistical analysis test; statistically significant differences were noted in the COVID-19 preventive behavior of populations between both study areas (*p*-value < 0.05).

The factors influencing the booster vaccination against COVID-19 in the post-COVID-19 period were identified in participants residing in the areas with the highest and lowest risk levels of environmental exposure in Thailand. The findings of our bivariate analysis determined the association between all the variables and the rate of administration of booster doses against COVID-19, with a *p*-value of 0.05 (Table 5). The results showed that the population age groups of 25 to 37 years (OR 2.05, 95% CI 1.39 to 3.10), 38 to 45 years (OR 2.17, 95% CI 1.43 to 3.30), and 46 to 53 years (OR 1.90, 95% CI 1.22 to 2.94) were significantly associated with booster dose. Subjects with a diploma degree (OR 3.41, 95% CI 2.45 to 4.76) and bachelor’s degree or higher (OR 4.90, 95% CI 2.23 to 10.78) were significantly more likely to receive a booster dose. People working in the government (OR 8.79, 95% CI 4.73 to 16.35) or private sectors (OR 2.74, 95% CI 1.27 to 3.65) were more likely to receive a booster dose. Subjects with monthly incomes of USD 144.1 to 287 (OR 1.80, 95% CI 1.24 to 2.62) or more than USD 287 (OR 2.94, 95% CI 2.04 to 4.24) were found to be significantly more likely to receive a booster dose to protect against COVID-19. Interestingly, a significant association with booster dose was found in the population residing in the areas with the lowest environmental exposure risk (OR = 1.34, 95% CI = 1.06 to 1.69). The best predictor of whether participants residing in the area with the highest and lowest risk levels for environmental exposure received a booster dose after the COVID-19 outbreak was identified using multivariate analysis. The result indicated that the working age groups (≥25-year-olds) were more likely to take the booster vaccine than the young age group: those aged 25 to 37 (AOR 1.74, 95% CI 1.06 to 2.85); aged 38 to 45 (AOR 2.24, 95% CI 1.31 to 3.83); aged 46 to 53 (AOR 2.25, CI 1.29–3.92); and aged ≥ 54 (AOR 2.21, CI 1.31 to 3.74). Participants with a diploma degree (AOR 2.39, 95% CI 1.53 to 3.72) and bachelor’s degree or higher (AOR 2.39, 95% CI 1.53 to 3.72) were reported to take the booster vaccine more frequently than participants with a lower education level. Participants working in full-time positions, including the government (AOR 6.09, 95% CI 3.19 to 11.63) and private sector (AOR 2.15, 95% CI 1.27 to 3.65), had higher rates of booster dose vaccination. Those with high monthly incomes between USD 144.1 and 287 and more than USD 287 significantly contributed to the number of booster vaccines received. Notably, populations residing in the area with the lowest risk of environmental exposure were 1.45 times more likely to accept a booster dose than those residing in the areas with the highest risk of environmental exposure (AOR 1.45, 95% CI 1.64 to 2.96).

## 4. Discussion

Using the environmental risk exposure index, the ten provinces ranked as having the highest and lowest number of cases of COVID-19 during the post-COVID-19 period were selected as potential areas of interest for this study [25]. Subjects in six locations, including the three provinces with the highest and the lowest levels of environmental risk exposures, were interviewed using structured questionnaires to compare the coverage and best predictors of booster dose vaccination against COVID-19. The results showed that the three areas with the highest risk of environmental exposure were Bangkok, Samut Prakan, and Samut Sakhon, which was similar to results of a related study conducted during the height of the COVID-19 pandemic, because of the high pollutant emissions in these locations [41]. The three lowest ranked risk areas, Mae Hong Son, Mukdahan, and Phichit Provinces, were selected for the study. The area with the lowest risk of environmental exposure was Mukdahan Province due to it having the lowest rates of lung cancer mortality and no days where it exceeded the limits set for PM10. McKay et al. [42] identified a significant association between air pollution and the incidence of lung cancer and lung adenocarcinoma [43]. In addition, air pollution and population density were the most important environmental factors increasing COVID-19 virus transmission across the world including in China, Italy, and Spain [10]. Hence, a study of the impact of environmental risk exposure in cities on COVID-19 transmission should be proposed to develop new strategies with a better understanding of environmental exposure mechanisms affecting virus transmission in Thailand.

After December 2019, when the first case of COVID-19 was identified in China, the WHO formally declared a global pandemic of COVID-19 in March 2020 [15]. Presently, over 4 million cases of COVID-19 have been reported and found in Thailand [33]. Laiphralpam et al.’s report [44] on COVID-19’s impact on Thailand found that the socio-economic issues caused affected the economy, unemployment, education, tourism, pollution, and mental health, while social stigma significantly increased during the two years of the pandemic. Subsequently, the Thai government formally announced the moving of its classification of COVID-19 to an endemic on 11 January 2022. However, the appropriate policy to prevent and control any pandemic and to decrease the impact of infection is vaccination [45]. Thus, the Thai government decided to promote new generation COVID-19 vaccines and increase the coverage of COVID-19 vaccination [24].

Andrews et al. [46] mentioned that the immunity level of two-dose COVID-19 vaccines declined over time and required a boost with a third, or more, doses of the vaccine to increase its preventive ability. In Thailand, the policy of recommending booster vaccines has been implemented since July 2021 to increase the immunity level of the COVID-19 vaccine with 80% of the population having had three doses, and 40% having had four doses by February 2023, because a high rate of COVID-19 re-infection was reported due to the new variants which evaded human immunity [47]. In particular, locations or cities with high levels of environmental pollutants increased the rate of the virus’s spread. Ousaneha et al. [34] reported that the area with the highest number of COVID-19 cases in Thailand was the capital city of Thailand, located in the Bangkok Metropolitan Region, which thus had the highest level of environmental exposure risk [41]. The promotion-based policy of increasing booster dose coverage will be continually implemented in areas with high environment pollutants to reduce the mortality and severity rates of COVID-19 during the post-pandemic period.

This is the first survey on the coverage rates of COVID-19 booster vaccines in populations living in the areas with the lowest and highest risk of environmental exposure in Thailand after the COVID-19 outbreak. Our results found that more than 63% of the Thai population received a booster dose (three or more vaccine doses) because of the Korean population’s trust with 69% of booster doses (three or more vaccine doses) because the population trusts that public health organizations have an important commitment to COVID-19 vaccination and updating booster vaccines during the post-pandemic era [48]. Moreover, it could be seen that the level of booster doses received declined during the post-COVID-19 period within the same study area, by 69.4% in one related study [25] to 51.1% in the current research due to a reduced fear of COVID-19 [49]. However, Sirison et al. [24] recommended that it was necessary to implement a policy of COVID-19 booster shots in Thai populations with benefits for economic and public health issues. 

For our multivariate analysis, working age, high education levels, being employed full-time in the government and private sector, a high monthly income, and living in the areas with the lowest risk of environmental exposure were the best predictors of COVID-19 booster vaccine uptake in Thai populations in the post-pandemic period. A synthetic review from Limbu and Bruce [50] reported that older people were consistently less hesitant than younger people to receive a booster dose because COVID-19 symptoms were directly experienced by older adult groups. 

Presently, groups with higher levels of education had a higher rate of the booster dose vaccine. According to a report in the *American Journal of Infection Control* [51], education level was a strong predictor of vaccine hesitancy. In this study, those who were full-time workers in the government and private sectors were more likely to take booster doses because being self-employed allows people more flexibility in their work environment and there is no requirement for vaccination from their workplaces [52]. Our findings revealed that the groups with high monthly incomes were more likely to receive COVID-19 booster vaccination. This result was similar to that of Al-Qerem et al. [53] who reported the high income portion of the population was associated with accepting the booster dose vaccine. The Thai government has provided a free-of-charge COVID-19 vaccine and booster dose to the public [25]. However, the cost of traveling to the vaccine center and lack of income from work were burdens to those receiving vaccines.

Surprisingly, populations living in the areas with the lowest risk level of environmental exposure were significantly more likely to take a COVID-19 booster dose after the COVID-19 outbreak. Daryanto et al. [39] indicated that the behavioral change of populations with respect to COVID-19 prevention came from the origin of COVID-19, human activity with environmental damage. Consequently, people were more likely to adopt good environmental behaviors after the COVID-19 outbreak. This finding was consistent with the result of the higher level of environmental behaviors noted in the areas with the lowest risk of environmental exposure (61.9%) compared to behaviors seen in the areas with the highest risk of environmental exposure (38.1%). Both situations, COVID-19 outbreak and environmental pollution, could signal a risk to humans and thus lead to the development and planning of protective behavior [54]. This finding was reflected in the no-booster-dose vaccine rates in the lowest risk area (41.2%) being lower than those in the areas with the highest risk of environmental exposure (58.8%). In the post-COVID-19 era, fewer participants negative for COVID-19 (39.1%) were found in the areas with the lowest risk of environmental exposure than those in the areas with the highest risk of environmental exposure (60.9%). The rates of COVID-19 vaccine booster acceptance within the lowest risk of environmental exposure were likely to be significantly affected in the post-pandemic period. Presumably, the results of environmental risk exposure were reversed concerning the rate of booster vaccines taken by different Thai populations after the COVID-19 pandemic.

Our research findings present an opportunity for policy makers to design suitable campaigns for developing preventive behaviors, increasing the populations’ perception of the association between environmental pollutants and global-scale epidemics [55]. As a result, the ongoing COVID-19 pandemic could have set off an alarm and initiated behavioral changes in terms of both the environment and health [39]. Specifically, continued preventive behaviors regarding the booster doses will be required for the management of COVID-19 [48]. Hence, a policy of routine immunization with an environmental awareness campaign will create a parallel leading to infectious disease control after a pandemic to achieve sustainable health development.

## 5. Conclusions

Our findings recommend routine immunization using the COVID-19 booster vaccine for continued individual protection after the pandemic, along with education on the concept of environmental risk exposure for Thai populations residing in urban locations. In the post-COVID-19 era, booster vaccinations against COVID-19 were less prevalent in the areas with the highest risk compared to those with the lowest risk of environmental exposure, as the lessened sense of panic and experience with COVID-19 infections in areas with high risks of environmental exposure created the highest number of COVID-19 cases in an area in Thailand. According to this finding, locations that had a risk of environmental exposure were directly impacted by preventive behaviors enforced by public health authorities for disease control. Therefore, the Thai government’s strategy could be promoted in parallel with environmental concerns. In the post-pandemic period, the COVID-19 booster vaccinations should be continually promoted for the at-risk group and easy access to vaccination centers should be provided at convenient times and locations. Furthermore, a health literacy aspect could support behavior changes and adaptation in creating an early warning process regarding new outbreaks of infectious disease. 

## Figures and Tables

**Table 1:** Risk profile of environmental exposure to COVID-19 in 20 provinces of Thailand.

Order	Province	Number of Cases of COVID-19	Total Value of Environmental Factors	Level of Environment Risk Exposure
Highest Number of Cases of COVID-19				
* 1	Bangkok	965,782	10.00	0.83
* 2	Samut Prakan	243,199	10.00	0.83
3	Chon Buri	229,716	6.00	0.50
* 4	Samut Sakhon	170,343	12.00	1.00
5	Nonthaburi	142,346	9.00	0.75
6	Nakhon Si Thammarat	126,883	5.00	0.42
7	Songkhla	103,443	3.00	0.25
8	Rayong	89,310	4.00	0.33
9	Pathum Thani	86,701	9.00	0.75
10	Ratchaburi	85,937	8.00	0.67
Lowest Number of Cases of COVID-19				
* 11	Mae Hong Son	6030	4.00	0.33
12	Lamphun	6260	6.00	0.50
13	Chai Nat	7178	7.00	0.58
14	Phayao	8899	9.00	0.75
* 15	Mukdahan	9942	3.00	0.25
16	Amnat Charoen	10,683	6.00	0.50
17	Phrae	11,341	7.00	0.58
* 18	Phichit	11,341	5.00	0.42
19	Chiang Rai	11,606	6.00	0.50
20	Lampang	13,214	6.00	0.50

Note: * = the provinces selected as the study area.

**Table 2:** Sociodemographic characteristics (*n* = 1315).

Characteristic	Category	Area [n (%)]			*p*-Value
		Low Risk	High Risk	Total	
Sex	Male	265 (47.0)	299 (53.0)	564 (42.9)	0.654
	Female	336 (44.7)	415 (55.3)	751 (57.1)	
Age	18–24	78 (50.3)	77 (49.7)	155 (11.8)	<0.001
	25–37	165 (40.7)	227 (53.0)	383 (29.1)	
	38–45	100 (37.2)	169 (62.8)	269 (20.5)	
	46–53	102 (50.7)	99 (43.3)	201 (15.3)	
	54+	165 (53.7)	142 (46.3)	307 (23.3)	
Education	Primary school	164 (52.2)	150 (47.8)	314 (23.9)	0.007
	Secondary school	239 (43.8)	307 (56.2)	546 (40.5)	
	Diploma degree	183 (45.4)	220 (54.6)	403 (30.6)	
	Bachelor’s degree and above	15 (28.8)	37 (71.2)	52 (4.0)	
Marital status	Single/divorced	282 (32.0)	599 (68.0)	881 (67.0)	<0.001
	Married	319 (73.5)	115 (26.5)	484 (33.0)	
Occupation	Self-employed	174 (42.5)	235 (57.5)	409 (31.1)	<0.001
	General employee	212 (46.8)	241 (53.2)	453 (34.4)	
	Student/not working	70 (49.0)	73 (51.0)	143 (10.9)	
	Government sector	110 (60.4)	72 (39.6)	182 (13.8)	
	Private sector	35 (27.3)	93 (72.7)	128 (9.7)	
Monthly income (USD)	Less than 144	101 (64.3)	56 (35.7)	157 (11.9)	<0.001
	144.1–287	273 (59.0)	190 (41.0)	463 (35.2)	
	More than 287	227 (32.7)	468 (67.3)	695 (52.9)	
COVID positive	No	198 (39.1)	308 (60.9)	506 (38.5)	<0.001
	Yes	403 (49.8)	406 (50.2)	809 (61.5)	
Source of COVID-19	Do not know	86 (39.1)	76 (46.9)	162 (21.8)	0.008
	Family member	107 (47.3)	119 (52.7)	226 (30.4)	
	Colleague	86 (45.7)	102 (54.3)	188 (25.3)	
	Expose to high-risk area	58 (34.7)	109 (65.3)	167 (22.5)	
Health insurance	No	506 (46.9)	574 (53.1)	1080 (82.1)	0.070
	Yes	95 (40.4)	140 (59.6)	235 (17.9)	

**Table 3:** Number of COVID-19 booster vaccines received.

Characteristic	Category	Area [n (%)]			*p*-Value
		Low Risk	High Risk	Total	
Number of vaccines received	1	4 (19.0)	17 (81.0)	21 (1.6)	0.001
	2	178 (42.2)	244 (57.8)	422 (32.1)	
	3	297 (52.0)	274 (48.0)	57.1 (43.4)	
	4	88 (41.1)	126 (58.9)	214 (16.3)	
	5	17 (36.2)	30 (63.8)	47 (3.6)	
Booster dose received	No	199 (41.2)	284 (58.8)	483 (36.7)	0.013
	Yes	402 (48.3)	430 (51.7)	832 (63.7)	

**Table 4:** Levels of COVID-19 impact, environmental concerns, and COVID-19 preventive behavior in the study areas.

Characteristic	Category	Area [n (%)]			*p*-Value
		Low Risk	High Risk	Total	
COVID-19 Impact					
Causal attribution	Low	376 (44.9)	461 (55.1)	837 (63.7)	0.452
	High	225 (47.1)	253 (52.9)	478 (36.3)	
Emotion	Low	372 (46.3)	432 (53.7)	804 (61.1)	0.606
	High	229 (44.8)	282 (52.2)	511 (38.9)	
Resilience	Low	406 (46.4)	469 (53.6)	875 (66.5)	0.475
	High	195 (44.3)	245 (55.7)	440 (33.5)	
Environmental Concern					
Environmental attitude	Poor	176 (46.4)	203 (53.6)	379 (28.8)	0.734
	Good	425 (45.4)	511 (57.6)	936 (71.2)	
Environmental behavior	Poor	398 (40.3)	589 (59.7)	987 (75.1)	<0.001
	Good	203 (61.9)	125 (38.1)	328 (25.9)	
COVID-19 Preventive Behavior					
Preventive behavior	Low	357 (43.0)	476 (57.0)	833 (63.4)	0.009

**Table 5:** Populations that received the COVID-19 booster dose living in areas with a high or low risk of environmental exposure.

Variable	Booster Dose Vaccination		Bivariate Analysis		Multivariate Analysis	
	No (%)	Yes (%)	COR ^a^ (95% CI)^c^	*p*-Value	AOR ^b^ (95% CI)^c^	*p*-Value
Gender						
Male	189 (35.1)	349 (64.9)	1			
Female	254 (34.5)	483 (65.5)	1.03 (0.81–1.30)	0.805		
Age						
18–24	72 (47.4)	80 (52.6)	1		1	
25–37	115 (30.5)	262 (69.5)	2.05 (1.39–3.01)	<0.001	1.74 (1.06–2.85)	0.028
38–45	76 (29.2)	184 (70.8)	2.17 (1.43–3.30)	<0.001	2.24 (1.31–3.83)	0.003
46–53	63 (32.1)	133 (67.9)	1.90 (1.22–2.94)	0.004	2.25 (1.29–3.92)	0.004
54+	117 (40.3)	173 (59.7)	1.33 (0.89–1.97)	0.157	2.21 (1.31–3.74)	0.003
Education						
Primary school	141 (47.2)	158 (52.8)	1		1	
Secondary school	211 (40.3)	312 (59.7)	1.32 (0.99–1.75)	0.058	1.39 (0.98–1.97)	0.060
Diploma degree	33 (20.7)	318 (79.3)	3.41 (2.45–4.76)	<0.001	2.39 (1.53–3.72)	<0.001
Bachelor’s degree or higher	8 (15.4)	44 (84.6)	4.90 (2.23–10.78)	<0.001	2.60 (1.10–6.40)	0.029
Marital status						
Single/divorced	289 (33.7)	569 (66.3)	1.15 (0.90–1.42)	0.307		
Married	154 (36.9)	263 (63.1)	1			
Occupation						
Self-employed	153 (38.7)	242 (61.3)	1		1	
General employee	190 (44.0)	242 (56.0)	0.80 (0.61–1.06)	0.126	0.93 (0.69–1.24)	0.631
Student/not working	64 (45.4)	77 (54.6)	0.76 (0.51–1.12)	0.168	1.50 (0.86–2.63)	0.148
Government sector	12 (6.7)	167 (93.3)	8.79 (4.73–16.35)	<0.001	6.09 (3.19–11.63)	<0.001
Private sector	24 (18.8)	104 (81.3)	2.74 (1.64–4.46)	<0.001	2.15 (1.27–3.65)	0.004
Income (USD)						
Less than 144	79 (53.4)	69 (46.6)	1		1	
144.1–287	174 (38.8)	274 (61.2)	1.80 (1.24–2.62)	0.002	1.76 (1.12–2.76)	0.013
More than 287	190 (28.0)	489 (72.0)	2.94 (2.04–4.24)	<0.001	1.73 (1.05–2.87)	0.031
COVID-19						
No	177 (36.6)	307 (63.4)	1			
Yes	266 (33.6)	525 (66.4)	0.81 (0.69–1.11)	0.284		
Insurance						
Yes	367 (35.2)	675 (64.8)	1			
No	76 (32.6)	157 (67.4)	1.12 (0.83–1.51)	0.451		
Area						
Low risk	182 (31.2)	402 (68.8)	1.34 (1.06–1.69)	0.014	1.45 (1.11–1.89)	0.006
High risk	261 (37.8)	430 (62.2)	1			
COVID-19 impact						
Causal attribution						
Low	282 (34.8)	528 (65.2)	1			
High	161 (34.7)	303 (65.3)	1.00 (0.78–1.27)	0.979		
Emotion						
Low	276 (35.4)	503 (64.6)	1			
High	167 (33.7)	324 (66.3)	0.08 (0.85–1.37)	0.520		
Resilience						
Low	289 (34.0)	560 (660.)	1			
High	154 (36.2)	272 (63.8)	0.92 (0.71–1.16)	0.455		
Environmental						
concern						
Environmental attitude						
Poor	123 (34.7)	231 (65.3)	1			
Good	320 (34.7)	601 (65.3)	1.00 (0.77–1.29)	1.00		
Environmental behavior						
Poor	333 (35.0)	619 (65.0)	1			
Good	110 (34.1)	213 (65.9)	1.01 (7.9–1.28)	0.903		
COVID-19 preventive behavior						
Poor	280 (34.9)	523 (65.1)	1			
Good	163 (34.5)	309 (65.5)	1.04 (0.79–1.35)	0.763		

Note: COR ^a^ = crude odds ratio; AOR ^b^ = adjusted odds ratio; and 95% CI ^c^ = 95% confidence interval.

## Data Availability

Requests to access the datasets should be directed to corresponding author.
